# The H9c2(2-1) cell-based sulforhodamine B assay is a non-animal alternative to evaluate municipal wastewater quality over time

**DOI:** 10.1007/s10661-023-12017-8

**Published:** 2023-10-31

**Authors:** Elsa T. Rodrigues, Eduarda Pereira, Paulo J. Oliveira, Miguel A. Pardal

**Affiliations:** 1 Centre for Functional Ecology, Associate Laboratory TERRA, Department of Life Sciences, University of Coimbra, Calçada Martim de Freitas, 3000-456 Coimbra, Portugal; 2 Department of Chemistry and CESAM/REQUIMTE, University of Aveiro, Campus de Santiago, 3810-193 Aveiro, Portugal; 3 Center for Neuroscience and Cell Biology, UC Biotech, University of Coimbra, Biocant Park, 3060-197 Cantanhede, Portugal

**Keywords:** Effluent toxicity testing, Environmental biomonitoring, Environmental health indicators, Temporal variability, H9c2(2-1) cells, Sulforhodamine B assay

## Abstract

The present study validates the potential of the *in vitro* H9c2(2-1) cell-based sulforhodamine B (SRB) assay to evaluate the temporal variability of wastewater quality. The impact of effluent disposal on water quality and the efficiency of the wastewater treatment process were also assessed. To correlate standard analytical method results with *in vitro* results, a total of 16 physicochemical parameters, such as nutrients, pH, chemical oxygen demand, total suspended solids and metals, were determined in both raw and treated wastewater samples. Results revealed that the H9c2(2-1) cell-based SRB assay has an enormous potential to evaluate municipal wastewater quality over time and to discriminate influent and effluent toxic characteristics, as well as for water quality monitoring and surveillance of the efficacy of treatment processes. Finally, the gathered results alerted to the impact of phosphates in a biological system, leading us to recommend the selection of this parameter as a potential environmental health indicator.

**Supplementary Information:**

The online version contains supplementary material available at 10.1007/s10661-023-12017-8.

## Intorudction

Wastewater outlet effluents, when inefficiently treated, might be major contributors to numerous water pollution problems, with negative consequences to aquatic life and human health (Aristi et al., 2015; Hamdhani et al., 2020). In addition, more than ever, the world faces increasing pressure on its water resources, and wastewater recycling and reuse are key subjects for rational management and research. Hence, the development of reliable methods to routinely assess wastewater toxicity is required to evaluate possible hazards to human health and guarantee the quality of treated wastewater (OSPAR Convention, 2000). Wastewater quality evaluation is conventionally based on physicochemical parameters, but increasing attention has been paid to their association with biological data due to the limitation of analytical methods to evaluate the characteristic mixture of pollutants present in wastewaters as a whole. Aquatic animal toxicity testing, which typically uses conventional assays with microalgae, *Daphnia magna* and fish (the whole animal or its eggs), is not compulsory for wastewater quality assessment in Europe and has been carried out on a voluntary basis. In the case of private companies, e.g. refineries, wastewater toxicity testing is only carried out to fulfil permit requirements, or at the request of regulators, and the most frequently reported tests use *D. magna* and fish eggs (https://www.concawe.eu/wp-content/uploads/2018/02/Rpt_18-2.pdf). Nevertheless, toxicity tests with vertebrate animals present key limitations, namely the use of animals as experimental models as being ethically not correct, demand high financial and expertise resources, and require licensed animal houses for the production of the animals or the eggs. Thus, addressing the limitations of both laboratory-based chemical methods and the use of animals as experimental models, several *in vitro* cell-based assays have been proposed to characterise wastewater toxicity in a biological system (Rodrigues et al., 2020), evaluate the efficacy of wastewater treatment and recycling processes (Shrivastava et al., 2017), or select the best disinfection method to prevent the spread of pathogens in a way that minimises outlet effluent toxicity (Dong et al., 2017).

We have previously shown that the rat cardiomyoblast H9c2(2-1) cell-based sulforhodamine B (SRB) colorimetric assay is suitable to quantitatively estimate the biological component of effluent toxicity (Rodrigues et al., 2020a) and as a promising *in vitro* method to replace fish lethal testing of municipal effluents, both in relative and absolute terms (Rodrigues et al., 2021). Since wastewater composition is highly variable as a function of time, day of the week and season, in a stepwise approach, the present study aims to confirm the potentiality of the H9c2(2-1) cell-based SRB assay to evaluate the temporal variability of municipal wastewater collected over a year. Confirming whether cell-based results reveal wastewater composition, a total of 16 physicochemical parameters were determined covering nutrients, pH, chemical oxygen demand (COD), total suspended solids (TSS) and metal elements. To assess the impact of effluent disposal on water quality, physicochemical results were compared with the specific discharge limits required by the Portuguese legislation for environmental protection against wastewater disposal in the water environment (Decree-law 236, 1998, *Annex XVIII*). Finally, the efficiency of the wastewater treatment process was evaluated as both raw influent and treated effluent samples were analysed.

The present study validates the use of a single bioassay for municipal wastewater testing over time, providing helpful information about the potential use of a non-animal assay as an efficient tool for environmental biomonitoring.

## Material and methods

### Characterisation of the wastewater treatment plant

The municipal wastewater treatment plant selected for the present study collects and treats hospital wastewater from an hospital with 1736 beds and 297,654 urgency admissions (data from 2018, https://www.chuc.min-saude.pt) and is also responsible for the treatment of the raw wastewater of 140,796 residents (Portuguese 2021 census). This wastewater treatment plant uses a pre-treatment of the influent consisting of three mechanical cleaning grilles and two desanders, two circular decanters for the primary treatment process and a secondary treatment composed of four percolating beds with rotary distributors and ventilation channels and two circular secondary decanters.

### Sampling procedures and sample preservation

The wastewater samples were collected monthly (inlet influents) and weekly (outlet effluents) during the year 2020, between Saturdays and Mondays, in a total of 11 influent (in January, no sample was collected) and 52 effluent samples. The time of grab sampling is reported in Table S1 (supplementary material). For sample collection, an amber glass container was completely filled (≈2.5 L) and transported to the laboratory (≈20 min), where it was immediately processed or refrigerated at 4 °C to be processed within 24 h. Nevertheless, pH was always measured on arrival at the laboratory. Then, for phosphates (PO_4_^3−^), nitrates (NO_3_^−^), silicates (Si) and ammonium (NH_4_^+^) determination, 500 mL of raw sample was frozen (−18 °C) in PET containers. For COD determination, 25 mL of raw sample was acidified to a pH below 2 with H_2_SO_4_ (Sigma-Aldrich 320501) and preserved at 4 °C in glass containers until analysis. For TSS determination, a well-mixed measured volume of the sample was filtered through a pre-weighed glass microfiber filter (Whatman GF/F 1825047) and placed in an oven until constant weight, to be further re-weighed. For metals (arsenic (As), iron (Fe), lead (Pb), cadmium (Cd), chromium (Cr), copper (Cu), manganese (Mn), mercury (Hg) and nickel (Ni)) determination, 120 mL of raw sample was acidified to pH below 2 with 65% HNO_3_ (Panreac 213255) and stored in a glass container. Possible cross-contamination was verified against triplicated blanks using ultra-pure water. The four glass containers used (one for the sample and three for blanks) were previously acid-washed overnight with 65% HNO_3_, thoroughly rinsed with ultra-pure water, dried before sample storage, and then preserved at 4 °C until analysis. Finally, for cell-based assays, 200 mL of filtered (Whatman GF/F 1825047) sample was deep-frozen (−80 °C) in glass containers for further lyophilisation (Martin Christ Alpha 1-2 Ldplus).

### Chemistry-based assessment

Total suspended solids (mg L^−1^) determination was assessed by the difference of glass microfiber filter weights (before and after sample filtration), whose mass increase was divided by the wastewater volume filtered. Determinations of PO_4_^3−^, NO_3_^−^, Si and NH_4_^+^, as well as COD, were performed by photometric analysis using a Palintest Photometer 7500 after calibration was verified (Palintest standards PT804) and using the photometer Phot 28 method for PO_4_^3−^ (mg PO_4_^3−^ L^−1^), the Phot 63 method for NO_3_^−^ (mg NO_3_^−^ L^−1^), the Phot 31 method for Si (mg Si L^−1^), the Phot 04 method for NH_4_^+^ (mg NH_4_^+^ L^−1^) and the Phot 81 method for COD (mg O_2_ L^−1^) determinations. The Palintest procedures were all performed following supplier instructions. Metal elemental analysis (except Hg) was determined using an inductively coupled plasma-mass spectrometer (ICP-MS) Thermo X Series. The ICP-MS was equipped with peristaltic pump, Burgener nebulizer and Ni cones. To ionise the sample molecules (sample flow ≈1 mL min^−1^), the equipment worked at 1400 W with an Argon flow of 13 L min^−1^ and an auxiliary gas flow of 0.8 L min^−1^. The calibration curve was made with multi-element standards diluted from certified standards for ICP analysis. Correlation coefficients under 0.999 were discarded and the error associated with each standard never exceeded 10%. The acceptable relative standard deviation between sample duplicates was < 5%. The quantification of Hg was carried out using cold vapour atomic fluorescence spectroscopy (CVAFS). The CVAFS was equipped with a 10.003 PSA cold vapour generator associated with a 10.023 Merlin PSA detector, and 2% of SnCl_2_ prepared in 10% of HCl was used as reducing agent. Calibration was performed using at least five acidified standards, which were prepared by dilution of a “BDH” Hg(NO_3_)_2_ standard solution of 1.0 g L^−1^. Confirming the calibration status of the equipment, a standard was analysed between every three samples to check for instrument drift. The acceptable relative standard deviation between sample triplicates was < 5%. The method detection limits and method quantitation limits by metal element were as follows: As (2.0 and 5.0 μg L^−1^), Fe (10 and 25 μg L^−1^), Pb (0.1 and 0.25 μg L^−1^), Cd (0.1 and 0.25 μg L^−1^), Cr (1.0 and 2.5 μg L^−1^), Cu (2.0 and 5.0 μg L^−1^), Mn (0.2 and 0.5 μg L^−1^), Hg (0.1 and 0.25 μg L^−1^) and Ni (1.0 and 2.5 μg L^−1^).

### H9c2(2-1)-based assays

H9c2(2-1) cells came from the ATCC cell bank (CRL-1446) and were grown in a sterile environment using a humidified atmosphere with 5% of CO_2_ at 37 °C (CO_2_ Unitherm, UniEquip). An adherent cell monolayer was cultured in dishes (VWR 734-2321) with filtered (Autofil 1102-RLS) DMEM-high glucose (Sigma-Aldrich D5648) culture medium adjusted to contain 1.8 g L^−1^ of sodium bicarbonate and supplemented with 10% of fetal bovine serum (Gibco 10270-106) and 1% of antibiotic-antimycotic (Gibco 15240-062), at pH 7.3.

Based on the stoichiometric ability of the SRB dye to bind to protein components of cells, the SRB assay was selected to evaluate cell growth inhibition potential of wastewater samples (Vichai & Kirtikara, 2006). To reduce possible cell passage effects, a maximum cell passage number of #20 was used in all the SRB assays performed. For that, H9c2(2-1) cells were plated the day prior to the assay at 10^4^ cells mL^−1^ density in 48-well plates (Corning 3548). To prepare the exposure solutions, 4.0 mL of cell culture medium was added to each lyophilised sample, and a sonicator (Branson Ultrasonics, 3510E-DTH) was used for 2 min to promote homogenisation. Then, in line with our two related studies (Rodrigues et al., 2021; Rodrigues et al., 2020a), in the highest concentration well, 400 μL of the well medium was removed and replaced with 900 μL of exposure solution, and then serial dilutions were applied, making a test concentration range of 35.2–4,500%. Three replicates prepared from independent cell cultures (true replicates) were maintained for each concentration, and four negative (cells with culture medium alone) and four positive (cells with 2% DMSO prepared in culture medium) controls were considered by replicate. Negative controls were used to establish the baseline, while positive controls were used to verify that the assay was run properly (e.g. effluent samples in theory should not be toxic). After a 24-h exposure time, cells were washed with phosphate buffer solution, dried, and then fixed at −18 °C with cold 1% of acetic acid prepared in methanol (Honeywell 34885). The fixative was removed and cells were stained for 60 min at room temperature using SRB solution (prepared in 1% of acetic acid in ultra-pure water) and excess of dye was removed by washing the wells with 1% of acetic acid prepared in ultra-pure water. Protein-bound dye was dissolved under gentle stirring using 10 mM Tris/base (Sigma-Aldrich T1503) at pH 10 and quantified from absorbance measurements (545 nm) using a microplate reader (BioTek Synergy HT).

### Validity criteria and statistical analysis

To ascertain reproducibility and as plate acceptance criteria, the coefficient of variation of the mean (CV, in percentage) was calculated for the negative controls (Iversen et al., 2012). CV was calculated by the equation:

$$\textrm{CV}\ \left(\%\right)=\left(\textrm{SD}/\sqrt{n}\right)/\textrm{arithmetic}\ \textrm{mean}\times 100$$

where SD is the standard deviation and *n* is the number of negative control wells per independent experiment. The acceptance criterion is CV ≤ 20%.

Non-linear regression analysis and curve fitting parameter were performed to calculate EC_50s_ (95% confidence interval). For that, absorbance SRB data were expressed as a fraction of the negative controls. Then, a four-parameter logistic regression after log-transformation of *x*-axis values was applied (GraphPad Prism 6 software). To validate the EC_50s_ results, the fitted concentration-response curves should have an *r*^2^ (coefficient of determination) ≥ 0.85, and the percent fitting error (%FE) of EC_50_ must be ≤ 40% (Beck et al., 2017). FE was calculated by the equation:

$$\%\textrm{FE}=\textrm{FE}\left(\textrm{LogEC}50\right)\times \textrm{Ln}(10)\times 100$$

where FE (LogEC_50_) is the standard error of LogEC_50_.

After verifying normality (Shapiro-Wilk test) and homogeneity (Levene’s test) assumptions and to statistically detect significant differences between SRB negative and positive controls, Student’s *t*-test (independent groups) was selected, and a significance threshold of 0.05 was considered (STATISTICA 7 software). This test was also used to evaluate the wastewater treatment process by statistically detecting significant differences between influent and effluent monthly data (for PO_4_^3−^, NO_3_^−^, Si, TSS, Fe, Mn and EC_50,24h_ data sets, logarithmic data transformation was previously performed).

In order to characterise the ability of the H9c2(2-1) cell-based SRB assay to assess the temporal variability of municipal wastewater, correlation coefficients between H9c2(2-1) EC_50,24h_ data and PO_4_^3−^, NO_3_^−^, Si, NH_4_^+^, COD, TSS, Fe, Pb, Mn and Ni were calculated. Daily rainfall data provided by the Portuguese Institute for Sea and Atmosphere were also considered. In line with the precautionary approach, correlations were only possible for COD (for influent data) since the missing value of November was completed with the maximum value determined during the year, as well as for Pb and Ni (for both influent and effluent data sets) as gaps (when values were below MQL) were completed with a value immediately below of MQLs: 0.24 μg L^−1^ for Pb and 2.4 μg L^−1^ for Ni. Since both influent and effluent H9c2(2-1) EC_50,24h_ data sets do not follow Gaussian distribution, the Spearman nonparametric correlation was selected to evaluate possible monotonic statistical relationships between cell-based data and the selected physicochemical variables (STATISTICA 7 software), and the Bonferroni correction was applied to deal with multiple testing by adjusting the significance level (Zar, 2010). Thus, a *P*-value of 0.0046 was used as the cut-off for significance.

## Results and discussion

Supplementary Tables S1 (nutrients, pH, COD and TSS) and S2 (metal elements) summarise the results of the physicochemical parameters for the wastewater samples. Regarding metals, As, Cd and Hg were always below the quantification limit of the method throughout the year, whereas all the others (Fe, Pb, Cr, Cu, Mn and Ni) occurred in a frequency that varied between 27.3 (Cr) and 100% (Fe and Mn) in the influent samples and between 17.3 (Cr) and 100% (Fe and Mn) in the effluent samples. The H9c2(2-1) cell-based SRB results showed that all the assays were accepted (validated), with the CV of negative controls never exceeding 4.4% (Table 1). Moreover, the positive controls (2% DMSO) always significantly decreased cell mass (*P* <0.05) in a mean percentage of 21.9 ± 4.8 (±standard deviation). The 24-h cell toxicity results are also presented in Table 1. In 9.6% of the cases, the effluent EC_50,24h_ determination, despite reportable, was not valid because of the low number of more extreme assay concentrations on the lower plateau, i.e. the sample presented low toxicity and, thus, a larger sample volume should have been lyophilised. Therefore, in those cases, a valid concentration-response curve was only possible after constraining the bottom of the curve to zero.

**Table 1:** Validity data and concentration-response relationship results of H9c2(2-1) SRB-based assays from municipal wastewater samples

Date	Influent				Effluent			
	CV Max (%)	FE (%)	Goodness of fit (*r*^2^)	EC_50,24h_ (95% CI) (%)	CV Max (%)	FE (%)	Goodness of fit (*r*^2^)	EC_50,24h_ (95% CI) (%)
Jan 4	-	-	-	-	1.8	4.7	0.975	2493 (2263–2748)*
Jan 11	-	-	-	-	1.6	7.4	0.987	1514 (1299–1766)
Jan 19	-	-	-	-	4.1	4.9	0.958	2777 (2506–3078)*
Jan 26	-	-	-	-	1.4	23	0.913	1842 (1139–2978)
Feb 2 or 4	1.0	4.7	0.984	365 (330.9–403.3)	1.8	6.3	0.942	2912 (2555–3320)*
Feb 9	-	-	-	-	2.4	22	0.941	1172 (733.7–1871)
Feb 16	-	-	-	-	2.8	11	0.942	1888 (1503–2373)
Feb 23	-	-	-	-	2.6	22	0.887	1810 (1145–2861)
March 1	2.4	4.9	0.986	630 (569.5–697.4)	1.9	18	0.910	1057 (731.6–1527)
March 8	-	-	-	-	2.2	12	0.937	941 (739.3–1198)
March 14	-	-	-	-	2.6	7.3	0.959	2161 (1858–2514)*
March 22	-	-	-	-	3.2	4.2	0.983	597 (547.8–651.5)
March 29	-	-	-	-	1.5	12	0.962	968 (747–1255)
April 6	2.0	22	0.921	775 (486.0–1235)	2.3	5.4	0.977	1279 (1142–1433)
April 13	-	-	-	-	2.9	8.1	0.967	760 (641.7–899.3)
April 19	-	-	-	-	2.7	10	0.926	919 (741.3–1138)
April 27	-	-	-	-	2.2	3.4	0.990	528 (491.6–567.0)
May 4	3.5	7.2	0.967	400 (344.4–464.2)	3.3	9.9	0.962	914 (743.8–1123)
May 11	-	-	-	-	1.5	10	0.926	644 (521.1–794.7)
May 18	-	-	-	-	1.4	11	0.929	480 (383.7–599.2)
May 25	-	-	-	-	1.6	7.3	0.971	493 (423.6–573.9)
May 30	-	-	-	-	1.7	11	0.973	801 (639.2–1003)
June 8	1.1	6.3	0.975	476 (417.5–542.5)	2.1	12	0.921	451 (351.7–578.1)
June 15	-	-	-	-	2.1	9.5	0.970	995 (816.7–1212)
June 22	-	-	-	-	4.4	11	0.937	996 (785.8–1263)
June 29	-	-	-	-	3.6	8.6	0.966	1116 (932.3–1335)
July 6	1.4	6.4	0.971	707 (619.1–807.3)	3.7	14	0.941	1098 (825.8–1460)
July 13	-	-	-	-	2.7	5.2	0.939	3681 (3305–4101)*
July 20	-	-	-	-	1.9	8.6	0.983	1021 (852.7–1221)
July 27	-	-	-	-	2.7	7.8	0.964	1910 (1623–2248)
August 3	0.9	11	0.952	519 (411.3–655.5)	0.83	32	0.946	1178 (602.5–2303)
August 8	-	-	-	-	2.8	15	0.893	851 (621.9–1165)
August 17	-	-	-	-	2.2	27	0.953	1364 (771.0–2412)
August 24	-	-	-	-	1.7	12	0.970	1055 (823.6–1351)
August 31	-	-	-	-	1.0	23	0.981	1422 (872.3–2320)
September 5	3.6	14	0.886	419 (314.5–558.5)	2.7	6.5	0.982	638 (556.8–731.3)
September 14	-	-	-	-	2.8	15	0.971	818 (594.4–1124)
September 21	-	-	-	-	1.9	9.4	0.931	1677 (1379–2039)
September 28	-	-	-	-	1.0	13	0.956	1030 (786.4–1349)
October 3	2.5	11	0.954	341 (268.2–433.1)	3.9	12	0.981	908 (702.4–1174)
October 12	-	-	-	-	1.6	4.2	0.986	962 (880.8–1050)
October 19	-	-	-	-	1.3	5.1	0.984	901 (809.8–1001)
October 26	-	-	-	-	1.2	16	0.910	1456 (1046–2027)
November 2	2.1	5.5	0.978	354 (315.4–396.6)	2.2	10	0.956	932 (753.8–1151)
November 9	-	-	-	-	1.9	8.3	0.980	832 (699.8–987.9)
November 16	-	-	-	-	1.9	6.0	0.972	652 (575.3–738.1)
November 23	-	-	-	-	1.8	9.4	0.983	800 (657.8–972.5)
November 30	-	-	-	-	0.8	5.6	0.983	479 (426.7–538.3)
December 7	2.2	9.9	0.943	1668 (1356–2053)	1.8	11	0.955	813 (640.4–1032)
December 14	-	-	-	-	1.9	7.9	0.973	979 (829.2–1155)
December 21	-	-	-	-	1.6	10	0.971	717 (585.9–876.8)
December 26	-	-	-	-	3.2	8.2	0.967	498 (419.3–591.4)

*CV* coefficient of variation of the mean of negative controls, *FE* fitting error, *CI* confidence interval

*After constraining the bottom of the curve to zero

Possibly due to the low number of data pairs (*N* = 11), correlations revealed that for influent data no significant relationships were observed between H9c2(2-1) results (EC_50,24h_) and the selected variables, whereas effluent H9c2(2-1) results covariated negatively with three variables: PO_4_^3−^, COD and TSS, and positively with one variable: Mn (Table 2). This means that toxicity EC_50,24h_ values decrease (indicating higher toxicity of the wastewater) whenever PO_4_^3−^, COD and TSS values increase, and that toxicity EC_50,24h_ values decrease when Mn decreases. The correlation results suggest that PO_4_^3−^ is a major contributor to biological impairment. In fact, many intracellular pathways utilise phosphate ions (inorganic phosphate) for important cellular reactions, and growing evidence links high phosphate levels to the increased risk of cardiovascular disease (recently reviewed by Zhou et al., 2021). Corroborating this, Brown and Razzaque (2016) have long pointed out that phosphate toxicity is a stealth biochemical stress factor for human health. Linking the environment and human health (the *One Health* approach), this parameter in wastewater could, therefore, be considered a suitable environmental health indicator. Regarding Mn, the determined levels were in line with its normal range in drinking water (recommended safety limit is 50 μg L^−1^, Decree-law 152, 2017), and thus a positive significant correlation was observed as it is known that this transition metal is an important cofactor nutrient and a structural component of many proteins, playing a vital role in the cellular metabolism. The same result would be expected for Fe, Cr, Cu and Ni, as they all serve important cellular roles (Andreini et al., 2008). However, the failure of a significant correlation with Fe is possibly because this metal element presented values above the Portuguese discharge limit in 29% of the sampled weeks (Table S2, supplementary material). This result is probably due to the fact that the selected wastewater treatment plant uses ferric chloride as an orthophosphate precipitation agent (technical information provided by the wastewater treatment plant). For Cr and Cu, no correlation analysis was performed due to their low occurrence frequency. The very low concentrations determined for Ni: maximum concentration of 7.4 μg L^−1^, which is even lower than the recommended safety limit of 20 μg L^−1^ for drinking water (Decree-law 152, 2017), could be a reason for the failure of a significant correlation. Correlation analysis also reveals that the effluent levels of NO_3_^−^, Si and NH_4_^+^ throughout the year do not impact the biological cell model selected for the present study, and neither does precipitation. The H9c2(2-1) cell-based SRB assay thus provided an estimate of the overall toxic burden of a mixture of pollutants present in municipal effluents over time. Gathered results corroborate previous findings that demonstrate the high sensitivity of H9c2(2-1) cells to environmental pollutants as pesticides (Rodrigues et al., 2015, 2019), pharmaceuticals (Bains et al., 2013; Rodrigues et al., 2020b), industrial chemicals (Han et al., 2017), charged polymers and heavy metals (Mohammad & Arfin, 2013), and toxins (Neves et al., 2020; Varela et al., 2020).

**Table 2:** Summary of the Spearman correlation results applied to H9c2(2-1)-based SRB results (EC_50,24h_) and abiotic variables of effluent samples collected weekly throughout 2020. Bold indicates significance (significance level at 0.0046)

H9c2(2-1)-based SRB results *vs*											
	PO_4_^3−^	NO_3_^−^	Si	NH_4_^+^	COD	TSS	Fe	Pb	Mn	Ni	pp
*r*	−0.526	−0.143	−0.165	−0.066	−0.610	−0.428	−0.034	0.179	0.485	0.071	−0.097
*P*	**< 0.0001**	0.3121	0.2440	0.6388	**< 0.0001**	**< 0.0046**	0.8105	0.2053	**< 0.0046**	0.6154	0.4943
*N*	52	52	52	52	52	52	52	52	52	52	52

*PO*_*4*_^*3−*^ phosphates, *NO*_*3*_^*−*^ nitrates, *Si* silicates, *NH*_*4*_^*+*^ ammonium, *COD* chemical oxygen demand, *TSS* total suspended solids, *Fe* iron, *Pb* lead, *Mn* manganese, *Ni* nickel, *pp* precipitation

The impact of effluent disposal on water quality was also studied, and according to the physicochemical results, several effluent data were non-compliant with the Portuguese standards, namely NO_3_^−^ (in 26.9% of the samples), NH_4_^+^ (88.5%), COD (9.6%), TSS (3.9%) and Fe (28.9%) (Tables S1 and S2, supplementary material). Since effluent NH_4_^+^ presented high levels during almost the whole year (non-compliance frequency of 88.5%), the biological nitrification process implemented in the wastewater treatment plant seems to require some kind of upgrade (e.g. properly sized lagoon aeration system) or a better control process (e.g. better monitoring of dissolved oxygen, biochemical oxygen demand, pH or temperature levels). To a smaller extent, denitrification (that converts NO_3_^−^ to nitrogen gas) seems to also need improvement. Based on the data obtained, this study highlights the need to develop technologies at wastewater treatment plant level so as to prevent pollutant output.

Despite the lack of synchronisation between influent and effluent samples due to the time of treatment in the wastewater treatment plant (the wastewater entering the system as influent comes out as effluent after 18–24 h), the H9c2(2-1) cell-based SRB assay was effective to discriminate influent and effluent toxic characteristics, as a significant difference was obtained by testing the two EC_50,24h_ data sets (*P* < 0.01), with effluents being 83.1% (mean value) less toxic to H9c2(2-1) cells than influents, thus demonstrating the success of this assay to evaluate the toxicity reduction of the wastewater treatment process. In December 7th, toxicity testing showed that the effluent (EC_50,24h_ = 813%) was more toxic than the influent (EC_50,24h_ = 1668%) (Table 1), which was possibly due to precipitation since it rained the night and the days before (data not shown), and it is known that rainwater can enter the municipal wastewater collection system and impair the performance of treatment facilities (Hummel et al., 2018), therefore impacting wastewater quality parameters (Suchowska-Kisielewicz & Nowogoński, 2021). In fact, five of the parameters evaluated—NO_3_^−^, Fe, Cr, Mn and Ni—showed higher values in the effluent than in the influent collected on that date (Tables S1 and S2, supplementary material). By comparing mean values of influent and effluent data, the wastewater treatment process allowed the expected increase of NO_3_^−^ due to nitrification in a percentage increase of 227.4%, and the effective reduction of PO_4_^3−^, NH_4_^+^, COD and TSS in a percentage decrease of 77.1, 54.5, 48.4 and 84.5%, respectively (all *P* values < 0,001). Both Si and pH remained unchanged (Si *P* value = 0.828, and pH *P* value = 0.316). Except for Fe and Mn, with an occurrence frequency of 100%, and for Ni which increased frequency, the occurrence frequency between influent and effluent decreased for Pb, Cr and Cu (see Table S2 of the supplementary material). When correspondent influent and effluent samples were compared, which was only possible when concentrations were above the MQL in both determinations, the results showed that Fe, Cu, Pb and Mn presented a significant difference (all *P* values ≤ 0.05), with an effective decrease of Cu (68% decrease) and Pb (63% decrease) levels and an increase of Fe (408% increase) and Mn (40% increase) levels, while Ni remained unchanged (*P* value = 0.965). Statistical analysis was not possible for Cr as only one correspondence was found.

The H9c2(2-1) cell-based SRB assay is thus a suitable bioanalytical tool for detection of non-specific toxicity and might be routinely applied for wastewater quality monitoring and for surveillance of the efficacy of treatment processes. Since high-quality water is becoming an increasingly scarce resource, and countries are including treated wastewater reuse as an essential dimension of water resources planning, new biomonitoring approaches that incorporate the 3R principles of *replacement*, *reduction* and *refinement* of animal testing are a step forward to ensure environmental and human safety in Europe. Nevertheless, advances in assay signal detection might improve H9c2 assay sensitivity, making current sample pre-concentration procedures unnecessary and reducing exposure times, as well as encompassing low-toxicity environmental pollutants. Hence, we advance that ultra-sensitive electrochemical biosensing detection might overcome the mentioned limitations.

## Conclusion

In a stepwise approach, we previously developed the H9c2(2-1) cell-based SRB assay for effluent toxicity assessment and as an alternative *in vitro* platform to investigate effluent toxicity in fish. Now, based on the results of the present study, we concluded that the H9c2(2-1) cell-based SRB assay also has an enormous potential to evaluate the temporal variability of municipal effluents, to discriminate influent and effluent toxic characteristics, and to evaluate effluent impact on the quality of the receiving water environment. Accordingly, this *in vitro* platform may present itself as a valuable proxy for effluent toxicity assessment in the context of animal alternatives, for surveillance of the efficacy of treatment processes, and as a bioanalytical tool for water quality monitoring. Nevertheless, the abovementioned assay still has limitations, e.g. a sample pre-concentration step is necessary for wastewater subject to analysis, which increases the total time of the assays. Thus, the next step is exploring ultra-sensitive devices such as microelectrode arrays that may reflect greater sensitivity to cell assays, which represents an important snapshot in time. Finally, the gathered results also alert to the impact of phosphates in a biological system, leading us to recommend the selection of this parameter as a suitable environmental health indicator.

### Supplementary information

ESM 1 (PDF 333 kb)

## Data Availability

The raw data are available as supplementary material.

## References

[CR1] Andreini C, Bertini I, Cavallaro G, Holliday GL, Thornton JM (2008). Metal ions in biological catalysis: From enzyme databases to general principles. Journal of Biological Inorganic Chemistry.

[CR2] Aristi I, von Schiller D, Arroita M, Barceló D, Ponsatí L, García-Galán MJ, Sabater S, Elosegi A, Acuña V (2015). Mixed effects of effluents from a wastewater treatment plant on river ecosystem metabolism: Subsidy or stress?. Freshwater Biology.

[CR3] Bains OS, Szeitz A, Lubieniecka JM, Cragg GE, Grigliatti TA, Riggs KW, Reid RE (2013). A correlation between cytotoxicity and reductase-mediated metabolism in cell lines treated with doxorubicin and daunorubicin. The Journal of Pharmacology and Experimental Therapeutics.

[CR4] Beck B, Chen Y-F, Dere W, Devanarayan V, Eastwood BJ, Farmen MW, Iturria SJ, Iversen PW, Kahl SD, Moore RA, Sawyer BD, Weidner J, Sittampalam GS, Coussens NP, Brimacombe K (2017). Assay operations for SAR Support. Assay Guidance manual.

[CR5] Brown RB, Razzaque MS (2016). Phosphate toxicity: A stealth biochemical stress factor?. Medical Molecular Morphology.

[CR6] Decree-law 152, 2017. Altera o regime da qualidade da água para consumo humano, transpondo as Diretivas 2013/51/EURATOM e 2015/1787. Diário da República 235/2017, Série I, 2017-12-07, pp 6555-6576. Available at: https://data.dre.pt/eli/dec-lei/152/2017/12/7/p/dre/pt/html

[CR7] Decree-law 236 (1998). Normas, critérios e objectivos de qualidade com a finalidade de proteger o meio aquático e melhorar a qualidade das águas em função dos seus principais usos. Diário da República, Série-IA.

[CR8] Dong S, Nguyen TH, Plewa MJ (2017). Comparative mammalian cell cytotoxicity of wastewater with elevated bromide and iodide after chlorination, chloramination, or ozonation. Journal of Environmental Sciences.

[CR9] Hamdhani H, Eppehimer DE, Bogan MT (2020). Release of treated effluent into streams: A global review of ecological impacts with a consideration of its potential use for environmental flows. Freshwater Biology.

[CR10] Han AA, Fabyanic EB, Miller JV, Prediger MS, Prince N, Mouch JA, Boyd J (2017). *In vitro* cytotoxicity assessment of a West Virginia chemical spill mixture involving 4-methylcyclohexanemethanol and propylene glycol phenyl ether. Environmental Monitoring and Assessment.

[CR11] Hummel MA, Berry MS, Stacey MT (2018). Sea level rise impacts on wastewater treatment systems along the U.S. coasts. Earth’s Future.

[CR12] Iversen PW, Beck B, Chen Y-F, Dere W, Devanarayan V, Eastwood BJ, Farmen MW, Iturria SJ, Montrose C, Moore RA, Weidner JR, Sittampalam GS, Sittampalam GS, Coussens NP, Brimacombe K (2012). HTS Assay validation.

[CR13] Mohammad F, Arfin T (2013). Cytotoxic effects of polystyrene-titanium-arsenate composite in cultured H9c2 cardiomyoblasts. Bulletin of Environmental Contamination and Toxicology.

[CR14] Neves RAF, Pardal MA, Nascimento SM, Oliveira PJ, Rodrigues ET (2020). Screening-level evaluation of marine benthic dinoflagellates toxicity using mammalian cell lines. Ecotoxicology and Environmental Safety.

[CR15] OSPAR Convention, 2000. The convention for the protection of the marine environment of the north-east Atlantic.

[CR16] Rodrigues ET, Nascimento SF, Moreno MJ, Oliveira PJ, Pardal MA (2020). Rat cardiomyocyte H9c2(2-1)-based sulforhodamine B assay as a promising *in vitro* method to assess the biological component of effluent toxicity. Research Journal of Environmental Sciences.

[CR17] Rodrigues ET, Pardal MA, Laizé V, Cancela ML, Oliveira PJ, Serafim TL (2015). Cardiomyocyte H9c2 cells present a valuable alternative to fish lethal testing for azoxystrobin. Environmental Pollution.

[CR18] Rodrigues ET, Pardal MA, Pereira E, Monteiro JF, Certal AC, Oliveira PJ (2021). H9c2(2-1)-based sulforhodamine B assay as a possible alternative in vitro platform to investigate effluent and metals toxicity on fish. Chemosphere.

[CR19] Rodrigues ET, Varela AT, Pardal MA, Oliveira PJ (2019). Cell-based assays seem not to accurately predict fish short-term toxicity of pesticides. Environmental Pollution.

[CR20] Rodrigues ET, Varela AT, Pardal MA, Sardão VA (2020). Cell-based assays as an alternative for the study of aquatic toxicity of pharmaceuticals. Environmental Science and Pollution Research.

[CR21] Shrivastava P, Naoghare PK, Gandhi D, Devi SS, Krishnamurthi K, Bafana A, Kashyap SM, Chakrabarti T (2017). Application of cell-based assays for toxicity characterization of complex wastewater matrices: Possible applications in wastewater recycle and reuse. Ecotoxicology and Environmental Safety.

[CR22] Suchowska-Kisielewicz M, Nowogoński I (2021). Influence of storms on the emission of pollutants from sewage into waters. Scientific Reports.

[CR23] Varela AT, Neves RAF, Nascimento SM, Oliveira PJ, Pardal MA, Rodrigues ET, Moreno AJ (2020). Mitochondrial impairment and cytotoxicity effects induced by the marine epibenthic dinoflagellate *Coolia malayensis*. Environmental Toxicology and Pharmacology.

[CR24] Vichai V, Kirtikara K (2006). Sulforhodamine B colorimetric assay for cytotoxicity screening. Nature Protocols.

[CR25] Zar JH (2010). Biostatistical analysis.

[CR26] Zhou C, Shi Z, Ouyang N, Ruan X (2021). Hyperphosphatemia and cardiovascular disease. Frontiers in Cell and Development Biology.

