# Supplementary material for: The H9c2(2-1) cell-based sulforhodamine B assay is a non-animal alternative to evaluate municipal wastewater quality over time
Source: Environ Monit Assess. 2023 Oct 31;195(11):1395. doi: 10.1007/s10661-023-12017-8 (PMC10618365; doi:10.1007/s10661-023-12017-8)
Supplement: Supplementary file 1 — (PDF 333 kb) [file 10661_2023_12017_MOESM1_ESM.pdf]

Supplementary material

The H9c2(2-1) cell-based sulforhodamine B assay is a non-animal alternative to evaluate municipal wastewater quality over time

Elsa T Rodrigues[§*], Eduarda Pereira[†], Paulo J Oliveira[∫], Miguel A Pardal[§]

[§]Centre for Functional Ecology, Department of Life Sciences, University of Coimbra, Calçada Martim de Freitas, 3000-456 Coimbra, Portugal. E-mail addresses: etrodrig@uc.pt (ET Rodrigues), mpardal@uc.pt (MA Pardal)

[†]Department of Chemistry and CESAM/REQUIMTE, University of Aveiro, Campus de Santiago, 3810-193 Aveiro, Portugal. E-mail address: eduper@ua.pt (E Pereira)

[∫]Center for Neuroscience and Cell Biology, UC Biotech, University of Coimbra, Biocant Park, 3060-197 Cantanhede, Portugal. E-mail address: pauloliv@cnc.uc.pt (PJ Oliveira)

*Corresponding author: ET Rodrigues

Table S1 Results of the physicochemical determined in laboratory for municipal wastewater samples, and emission limit values (ELV, mg L^{-1}) for the effluent samples according to the Portuguese legislation (Decree-Law 236, 1998, *Annex XVIII*). Bold indicates non-compliance with the Portuguese standards.

Date (time of collection)	Influent								Effluent						
	PO_4^{3-} (mg L^{-1})	NO_3^- (mg L^{-1})	Si (mg L^{-1})	NH_4^+ (mg L^{-1})	pH	COD (mg O_2 L^{-1})	TSS (mg L^{-1})		PO_4^{3-} (mg L^{-1})	NO_3^- (mg L^{-1})	Si (mg L^{-1})	NH_4^+ (mg L^{-1})	pH	COD (mg O_2 L^{-1})	TSS (mg L^{-1})
								ELV	-	50	-	10	6-9	150	60
Jan. 4 (10 h)	-	-	-	-	-	-	-		1.50	20.4	11	**27.0**	8.43	72	10.2
Jan. 11 (11 h)	-	-	-	-	-	-	-		1.80	21.2	6.5	**62.0**	7.70	88	20.4
Jan. 19 (11 h)	-	-	-	-	-	-	-		2.37	15.3	17	**21.0**	7.21	72	13.6
Jan. 26 (11:15 h)	-	-	-	-	-	-	-		1.72	17.2	17	**23.0**	7.17	67	14.0
Feb. 2 (11:30 h)	-	-	-	-	-	-	-		1.82	15.7	6.5	**45.0**	8.98	64	24.4
Feb. 4 (16 h)	6.43	5.80	3.4	45.0	7.05	210	183		-	-	-	-	-	-	-
Feb. 9 (11:15 h)	-	-	-	-	-	-	-		1.52	25.6	5.5	**55.0**	7.31	87	37.6
Feb. 16 (11 h)	-	-	-	-	-	-	-		1.72	13.6	2.8	**30.0**	7.69	78	21.3
Feb. 23 (11 h)	-	-	-	-	-	-	-		2.08	16.0	17	**34.0**	7.41	87	24.2
Mar. 1 (I: 11:20 h/E: 11 h)	8.70	7.36	19	45.0	8.06	162	247		3.20	19.9	6.0	**21.0**	7.40	72	27.0
Mar. 8 (11 h)	-	-	-	-	-	-	-		2.20	14.6	3.6	9.9	7.37	96	20.0
Mar. 14 (11 h)	-	-	-	-	-	-	-		2.97	23.0	10	7.4	7.51	110	18.6
Mar. 22 (14:15 h)	-	-	-	-	-	-	-		2.17	34.4	19	8.5	7.31	80	21.2
Mar. 29 (14 h)	-	-	-	-	-	-	-		3.53	35.8	4.4	**10.8**	7.35	80	23.2
Apr. 6 (I: 9:30 h/E: 9 h)	4.67	7.72	11	10.3	7.63	120	65.3		3.27	23.0	2.6	5.5	7.21	120	35.2
Apr. 13 (9:15 h)	-	-	-	-	-	-	-		7.80	31.1	6.0	**10.1**	7.42	115	17.6
Apr. 19 (15:15 h)	-	-	-	-	-	-	-		7.07	38.9	9.0	6.5	7.54	79	20.3
Apr. 27 (9:30 h)	-	-	-	-	-	-	-		7.40	39.8	12	7.5	7.48	92	18.7
May 4 (I: 9:30 h/E: 9:15 h)	8.17	6.34	7.0	57.0	7.65	230	176		4.20	22.0	8.0	**48.0**	7.23	108	16.0
May 11 (9:15 h)	-	-	-	-	-	-	-		3.60	37.4	10	**12.6**	7.33	110	29.2
May 18 (8:45 h)	-	-	-	-	-	-	-		2.63	24.0	8.5	**18.0**	7.63	98	19.1
May 25 (9:15 h)	-	-	-	-	-	-	-		3.53	**65.1**	17	**21.2**	7.56	115	27.3
May 30 (15:45 h)	-	-	-	-	-	-	-		3.30	**75.7**	4.0	**17.0**	7.33	72	25.4
June 8 (I: 9:30 h/E: 9:15 h)	40.0	21.7	5.0	74.0	8.00	240	224		12.0	**60.0**	5.5	**22.0**	6.75	108	26.4
June 15 (9:45 h)	-	-	-	-	-	-	-		2.50	**79.2**	3.4	**15.0**	6.61	74	22.8
June 22 (9:15 h)	-	-	-	-	-	-	-		2.90	**73.7**	4.4	**18.0**	7.16	94	22.8
June 29 (12 h)	-	-	-	-	-	-	-		2.90	**78.6**	3.4	**17.0**	7.40	76	17.6
July 6 (I: 9:15 h/E: 8:45 h)	15.5	9.53	2.6	62.0	7.33	210	204		1.90	**64.6**	4.0	**19.0**	7.16	84	14.0
July 13 (9:15 h)	-	-	-	-	-	-	-		1.50	45.0	3.8	**19.0**	7.21	74	9.6
July 20 (9:15 h)	-	-	-	-	-	-	-		5.10	40.5	2.0	**15.0**	7.13	**155**	**73.0**
July 27 (9 h)	-	-	-	-	-	-	-		1.30	**84.8**	3.2	**10.0**	7.05	58	11.6

Date (time of collection)	Influent								Effluent						
	PO_4^{3-} (mg L^{-1})	NO_3^- (mg L^{-1})	Si (mg L^{-1})	NH_4^+ (mg L^{-1})	pH	COD (mg O_2 L^{-1})	TSS (mg L^{-1})		PO_4^{3-} (mg L^{-1})	NO_3^- (mg L^{-1})	Si (mg L^{-1})	NH_4^+ (mg L^{-1})	pH	COD (mg O_2 L^{-1})	TSS (mg L^{-1})
								ELV	-	50	-	10	6-9	150	60
Aug. 3 (I: 9:15 h/E: 8:45 h)	17.0	13.7	5.0	45.0	7.44	195	168		1.30	**60.5**	3.4	**10.0**	7.18	66	15.2
Aug. 8 (10 h)	-	-	-	-	-	-	-		3.50	38.9	3.8	**18.0**	7.35	115	28.0
Aug. 17 (10 h)	-	-	-	-	-	-	-		2.90	**67.1**	4.0	**10.0**	7.25	98	22.0
Aug. 24 (9:30 h)	-	-	-	-	-	-	-		1.60	**73.9**	4.0	**12.0**	7.19	76	14.4
Aug. 31 (9:30 h)	-	-	-	-	-	-	-		4.50	**50.5**	4.2	**14.0**	7.22	66	15.2
Sep. 5 (I: 9 h/E: 9:15 h)	9.70	8.20	4.4	90.0	7.14	175	99.0		10.5	39.6	4.6	**17.0**	7.43	120	26.0
Sep. 14 (9:30 h)	-	-	-	-	-	-	-		1.70	42.2	3.6	**23.0**	7.32	88	16.4
Sep. 21 (9:30 h)	-	-	-	-	-	-	-		1.30	48.8	3.2	**11.0**	7.24	76	18.4
Sep. 28 (14:45 h)	-	-	-	-	-	-	-		2.10	49.3	3.2	**18.0**	7.33	72	18.4
Oct. 3 (I: 9:15 h/E: 9:30 h)	22.0	9.93	4.8	55.0	7.77	195	72.5		3.10	**69.3**	21	**15.0**	7.35	80	16.4
Oct. 12 (9:30 h)	-	-	-	-	-	-	-		3.50	19.9	3.8	**37.0**	7.60	140	50.8
Oct. 19 (9 h)	-	-	-	-	-	-	-		2.90	7.4	7.0	**68.0**	7.49	130	47.6
Oct. 26 (9:15 h)	-	-	-	-	-	-	-		1.60	34.5	3.0	**17.0**	7.32	70	39.2
Nov. 2 (I: 9 h/E: 9:30 h)	19.5	10.3	5.0	44.0	8.04	-	257		4.80	31.5	4.2	**26.0**	7.41	140	27.2
Nov. 9 (9 h)	-	-	-	-	-	-	-		2.00	49.0	3.8	**25.0**	7.48	92	28.8
Nov. 16 (8:45 h)	-	-	-	-	-	-	-		2.60	32.5	4.6	**44.0**	7.58	120	37.6
Nov. 23 (9:15 h)	-	-	-	-	-	-	-		2.00	**85.2**	4.4	**30.0**	7.55	110	20.4
Nov. 30 (9:45 h)	-	-	-	-	-	-	-		6.20	14.1	4.8	**27.0**	7.80	**185**	**64.0**
Dec. 7 (I: 9:30 h/E: 10 h)	8.50	18.2	4.6	40.0	7.79	185	107		1.40	38.3	3.6	**27.0**	7.55	108	25.0
Dec. 14 (17 h)	-	-	-	-	-	-	-		2.40	36.1	4.4	**39.0**	7.69	**155**	37.6
Dec. 21 (11:30 h)	-	-	-	-	-	-	-		1.30	42.3	4.4	**46.0**	7.72	**200**	30.0
Dec. 26 (10:30 h)	-	-	-	-	-	-	-		8.90	32.4	2.4	**52.0**	7.69	**160**	40.0
Maximum value	40	21.7	19	90.0	8.06	240	257		12.0	85.2	21	68.0	8.98	200	73.0
Mean value	14.6	10.8	6.5	51.6	7.63	192	164		3.34	40.8	6.51	23.5	7.42	99	25.4
Non-compliance frequency (%)	-	-	-	-	-	-	-		-	26.9	-	88.5	-	9.6	3.9

PO_4^{3-}, phosphates; NO_3^-, nitrates; Si, silicates; NH_4^+, ammonium; COD, chemical oxygen demand; TSS, total suspended solids

Table S2 Results (μg L^{-1}) of the metal elements determined in laboratory for municipal wastewater samples, method quantitation limits (MQL, μg L^{-1}), and emission limit values (ELV, μg L^{-1}) for effluent samples according to the Portuguese legislation (Decree-Law 236, 1998, *Annex XVIII*). Bold indicates non-compliance with the Portuguese standards.

Date (time of collection)		Influent										Effluent								
		As	Fe	Pb	Cd	Cr	Cu	Mn	Hg	Ni		As	Fe	Pb	Cd	Cr	Cu	Mn	Hg	Ni
	MQL	5.0	25	0.25	0.25	2.5	5.0	0.5	0.25	2.5	MQL	5.0	25	0.25	0.25	2.5	5.0	0.5	0.25	2.5
											ELV	1000	2000	1000	200	2000	1000	2000	50	2000
Jan. 4 (10 h)		-	-	-	-	-	-	-	-	-		<MQL	1076	0.55	<MQL	<MQL	<MQL	64	<MQL	5.3
Jan. 11 (11 h)		-	-	-	-	-	-	-	-	-		<MQL	1593	<MQL	<MQL	<MQL	6.1	68	<MQL	3.9
Jan. 19 (11 h)		-	-	-	-	-	-	-	-	-		<MQL	1299	1.71	<MQL	<MQL	<MQL	61	<MQL	3.5
Jan. 26 (11:15 h)		-	-	-	-	-	-	-	-	-		<MQL	1317	0.78	<MQL	<MQL	<MQL	57	<MQL	5.4
Feb. 2 (11:30 h)		-	-	-	-	-	-	-	-	-		<MQL	**2297**	1.0	<MQL	<MQL	5.4	67	<MQL	3.7
Feb. 4 (16 h)		<MQL	340	3.6	<MQL	3.5	19	32	<MQL	2.5		-	-	-	-	-	-	-	-	-
Feb. 9 (11:15 h)		-	-	-	-	-	-	-	-	-		<MQL	**2180**	0.51	<MQL	<MQL	5.4	80	<MQL	3.6
Feb. 16 (11 h)		-	-	-	-	-	-	-	-	-		<MQL	1980	0.56	<MQL	<MQL	<MQL	58	<MQL	4.3
Feb. 23 (11 h)		-	-	-	-	-	-	-	-	-		<MQL	**2437**	2.9	<MQL	<MQL	<MQL	49	<MQL	4.0
Mar. 1 (I: 11:20 h/E: 11 h)		<MQL	690	2.0	<MQL	3.5	24	40	<MQL	1.7		<MQL	**3650**	<MQL	<MQL	130	6.0	68	<MQL	7.4
Mar. 8 (11 h)		-	-	-	-	-	-	-	-	-		<MQL	**2335**	2.0	<MQL	<MQL	<MQL	57	<MQL	3.1
Mar. 14 (11 h)		-	-	-	-	-	-	-	-	-		<MQL	**2318**	2.3	<MQL	<MQL	<MQL	49	<MQL	5.1
Mar. 22 (14:15 h)		-	-	-	-	-	-	-	-	-		<MQL	**2282**	0.45	<MQL	<MQL	<MQL	51	<MQL	2.8
Mar. 29 (14 h)		-	-	-	-	-	-	-	-	-		<MQL	**3007**	1.2	<MQL	<MQL	<MQL	44	<MQL	3.1
Apr. 6 (I: 9:30 h/E: 9 h)		<MQL	149	0.56	<MQL	<MQL	7.0	28	<MQL	<MQL		<MQL	1265	0.63	<MQL	<MQL	5.3	32	<MQL	2.6
Apr. 13 (9:15 h)		-	-	-	-	-	-	-	-	-		<MQL	905	<MQL	<MQL	<MQL	5.6	28	<MQL	<MQL
Apr. 19 (15:15 h)		-	-	-	-	-	-	-	-	-		<MQL	834	0.50	<MQL	<MQL	6.8	28	<MQL	3.3
Apr. 27 (9:30 h)		-	-	-	-	-	-	-	-	-		<MQL	341	<MQL	<MQL	<MQL	5.4	25	<MQL	3.0
May 4 (I: 9:30 h/E: 9:15 h)		<MQL	355	2.3	<MQL	<MQL	19	38	<MQL	4.9		<MQL	1012	<MQL	<MQL	<MQL	<MQL	48	<MQL	3.2
May 11 (9:15 h)		-	-	-	-	-	-	-	-	-		<MQL	1650	<MQL	<MQL	<MQL	<MQL	37	<MQL	3.1
May 18 (8:45 h)		-	-	-	-	-	-	-	-	-		<MQL	1496	0.63	<MQL	<MQL	6.1	32	<MQL	1.9
May 25 (9:15 h)		-	-	-	-	-	-	-	-	-		<MQL	1789	0.35	<MQL	<MQL	<MQL	32	<MQL	3.8
May 30 (15:45 h)		-	-	-	-	-	-	-	-	-		<MQL	1944	<MQL	<MQL	<MQL	<MQL	38	<MQL	3.1
June 8 (I: 9:30 h/E: 9:15 h)		<MQL	479	1.7	<MQL	14	20	35	<MQL	7.3		<MQL	1227	0.33	<MQL	<MQL	6.1	26	<MQL	2.7
June 15 (9:45 h)		-	-	-	-	-	-	-	-	-		<MQL	1127	<MQL	<MQL	<MQL	<MQL	33	<MQL	<MQL
June 22 (9:15 h)		-	-	-	-	-	-	-	-	-		<MQL	1162	0.50	<MQL	<MQL	<MQL	33	<MQL	<MQL
June 29 (12 h)		-	-	-	-	-	-	-	-	-		<MQL	1379	0.35	<MQL	<MQL	5.2	31	<MQL	2.7
July 6 (I: 9:15 h/E: 8:45 h)		<MQL	242	0.91	<MQL	<MQL	15	27	<MQL	<MQL		<MQL	944	<MQL	<MQL	<MQL	<MQL	32	<MQL	<MQL
July 13 (9:15 h)		-	-	-	-	-	-	-	-	-		<MQL	1942	0.47	<MQL	<MQL	<MQL	46	<MQL	3.7
July 20 (9:15 h)		-	-	-	-	-	-	-	-	-		<MQL	**7147**	2.1	<MQL	3.4	11	74	<MQL	6.0
July 27 (9 h)		-	-	-	-	-	-	-	-	-		<MQL	976	<MQL	<MQL	<MQL	<MQL	39	<MQL	<MQL

Date (time of collection)		Influent										Effluent								
		As	Fe	Pb	Cd	Cr	Cu	Mn	Hg	Ni		As	Fe	Pb	Cd	Cr	Cu	Mn	Hg	Ni
	MQL	5.0	25	0.25	0.25	2.5	5.0	0.5	0.25	2.5	MQL	5.0	25	0.25	0.25	2.5	5.0	0.5	0.25	2.5
											ELV	1000	2000	1000	200	2000	1000	2000	50	2000
Aug. 3 (I: 9:15 h/E: 8:45 h)		<MQL	201	2.3	<MQL	<MQL	12	19	<MQL	<MQL		<MQL	1102	0.33	<MQL	<MQL	<MQL	41	<MQL	<MQL
Aug. 8 (10 h)		-	-	-	-	-	-	-	-	-		<MQL	1582	<MQL	<MQL	<MQL	<MQL	42	<MQL	3.0
Aug. 17 (10 h)		-	-	-	-	-	-	-	-	-		<MQL	1319	0.28	<MQL	<MQL	<MQL	43	<MQL	2.9
Aug. 24 (9:30 h)		-	-	-	-	-	-	-	-	-		<MQL	826	<MQL	<MQL	<MQL	<MQL	43	<MQL	<MQL
Aug. 31 (9:30 h)		-	-	-	-	-	-	-	-	-		<MQL	639	<MQL	<MQL	<MQL	<MQL	40	<MQL	3.3
Sep. 5 (I: 9 h/E: 9:15 h)		<MQL	66	<MQL	<MQL	<MQL	<MQL	24	<MQL	<MQL		<MQL	747	<MQL	<MQL	<MQL	<MQL	30	<MQL	5.0
Sep. 14 (9:30 h)		-	-	-	-	-	-	-	-	-		<MQL	1479	<MQL	<MQL	<MQL	<MQL	47	<MQL	3.6
Sep. 21 (9:30 h)		-	-	-	-	-	-	-	-	-		<MQL	1134	<MQL	<MQL	<MQL	<MQL	48	<MQL	<MQL
Sep. 28 (14:45 h)		-	-	-	-	-	-	-	-	-		<MQL	1002	0.31	<MQL	<MQL	<MQL	40	<MQL	3.9
Oct. 3 (I: 9:15 h/E: 9:30 h)		<MQL	226	0.40	<MQL	<MQL	8.5	30	<MQL	4.4		<MQL	838	<MQL	<MQL	<MQL	<MQL	28	<MQL	4.2
Oct. 12 (9:30 h)		-	-	-	-	-	-	-	-	-		<MQL	**2882**	<MQL	<MQL	2.6	5.9	61	<MQL	6.1
Oct. 19 (9 h)		-	-	-	-	-	-	-	-	-		<MQL	1405	0.32	<MQL	<MQL	5.5	43	<MQL	4.8
Oct. 26 (9:15 h)		-	-	-	-	-	-	-	-	-		<MQL	1459	0.31	<MQL	<MQL	<MQL	37	<MQL	2.9
Nov. 2 (I: 9 h/E: 9:30 h)		<MQL	350	1.4	<MQL	<MQL	23	31	<MQL	<MQL		<MQL	1884	1.1	<MQL	<MQL	7.4	45	<MQL	<MQL
Nov. 9 (9 h)		-	-	-	-	-	-	-	-	-		<MQL	**2001**	0.68	<MQL	<MQL	<MQL	47	<MQL	3.1
Nov. 16 (8:45 h)		-	-	-	-	-	-	-	-	-		<MQL	**2073**	0.50	<MQL	3.8	6.3	42	<MQL	3.8
Nov. 23 (9:15 h)		-	-	-	-	-	-	-	-	-		<MQL	1597	<MQL	<MQL	<MQL	<MQL	38	<MQL	3.9
Nov. 30 (9:45 h)		-	-	-	-	-	-	-	-	-		<MQL	1823	1.1	<MQL	4.6	10	38	<MQL	4.5
Dec. 7 (I: 9:30 h/E: 10 h)		<MQL	214	0.87	<MQL	<MQL	9.2	26	<MQL	<MQL		<MQL	1849	0.47	<MQL	2.8	<MQL	43	<MQL	3.6
Dec. 14 (17 h)		-	-	-	-	-	-	-	-	-		<MQL	**2327**	0.91	<MQL	3.0	6.2	40	<MQL	3.5
Dec. 21 (11:30 h)		-	-	-	-	-	-	-	-	-		<MQL	**2242**	0.47	<MQL	2.5	<MQL	41	<MQL	3.8
Dec. 26 (10:30 h)		-	-	-	-	-	-	-	-	-		<MQL	**2475**	0.65	<MQL	3.7	5.2	45	<MQL	4.0
Maximum value (μg L^{-1})		-	690	3.6	-	14	24	40	-	7.3		-	7147	2.9	-	130	11	80	-	7.4
Mean value (μg L^{-1})		-	301	1.6	-	7	16	30	-	4.2		-	1723	0.83	-	17	6.4	44	-	3.8
Occurrence frequency (%)		0	100	90.9	0	27.3	90.9	100	0	45.5		0	100	63.5	0	17.3	36.5	100	0	82.7
Non-compliance frequency (%)		-	-	-	-	-	-	-	-	-		0	28.9	0	0	0	0	0	0	0

As, arsenic; Fe, iron; Pb, lead; Cd, cadmium; Cr, chromium; Cu, copper; Mn, manganese; Hg, mercury; Ni, nickel
